# Remote Interrogation of WDM Fiber-Optic Intensity Sensors Deploying Delay Lines in the Virtual Domain

**DOI:** 10.3390/s130505870

**Published:** 2013-05-07

**Authors:** David Sánchez Montero, Carmen Vázquez

**Affiliations:** Departamento de Tecnología Electrónica, Universidad Carlos III de Madrid, Avda. de la Universidad, 30, 28911 Leganés, Madrid, Spain; E-Mail: cvazquez@ing.uc3m.es

**Keywords:** optical sensing, passive remote sensing, self-referencing, virtual delay line

## Abstract

In this work a radio-frequency self-referencing WDM intensity-based fiber-optic sensor operating in reflective configuration and using virtual instrumentation is presented. The use of virtual delay lines at the reception stage, along with novel flexible self-referencing techniques, and using a single frequency, avoids all-optical or electrical-based delay lines approaches. This solution preserves the self-referencing and performance characteristics of the proposed WDM-based optical sensing topology, and leads to a more compact solution with higher flexibility for the multiple interrogation of remote sensing points in a sensor network. Results are presented for a displacement sensor demonstrating the concept feasibility.

## 1. Introduction

Intensity fiber-optic sensors (FOS) provide an optical modulation signal as the measurement and use different self-referencing techniques to avoid noise errors from undesirable intensity fluctuations or variation in losses non-correlated to the sensor modulation. They have been proved to be easily integrated in wavelength-division multiplexing (WDM) networks, including those based on fiber Bragg gratings (FBGs), and have been demonstrated to provide an effective and compact strategy to operate in reflective configuration [1] for exploiting fiber links and for remotely addressing multiple sensing points with a single fiber lead [2,3] instead of spatial multiplexing deployments [4].

Configurations providing self-referencing techniques have been a research topic during the last years. The use of all-optical resonant structures as the basis of a self-referencing intensity type sensor has been widely discussed in the literature. Schemes based on a Fabry-Perot resonant structure [5], Michelson [6], Sagnac [7,8], and ring resonators [9–11], with fiber delay coils [1,12] are reported. For instance, in the work reported in Reference [1] identical fiber coils of 450 m were emplaced at each sensing point, with the requirement of being of identical length to share the two modulation frequencies at the transmission stage for all the sensors, otherwise the point of operation of the measurement technique would be different for each sensor, which is not a desirable situation.

Lately, the long fiber coils were replaced with electrical filters at the reception stage of the remote sensor network [13]. This solution provided arbitrary modulation frequencies, compact sensing points and flexibility in the operation of the sensor network. In addition, the optical power modulation from the remote sensing point could be related to the coefficients of the filter structure thus encoding the filter response either in amplitude or in phase, and then performing self-referencing measurements. Furthermore, a Coarse WDM (CWDM) reflective star sensor network topology for multiplexing and interrogation of *N* quasi-distributed self-referencing remote sensing points, using two electrical phase-shifts per sensor for flexibility purposes, was recently studied [14]. Two measurement parameters were defined, one based on phase measurements and another based on amplitude measurements. This electro-optical self-referencing solution was verified by modulating the light injected into the network and using a lock-in amplifier and electrical phase-shifters at the reception stage. On the other hand, it was recently reported a virtual delay line deployment [15], but in a Mach-Zehnder interferometric topology in which the optical source was modulated with two different frequencies and based on a power-splitting topology rather than in a WDM approach. The self-referencing parameter was defined as the ratio of voltage values of the optical output sinusoidal wave at a previously defined non-constructive and constructive interference frequency, respectively.

In this work, the feasibility for enhancing the automation of the interrogation, by working in the virtual domain, of remote intensity-based optical sensors operating in reflective configuration and deployed in a radio-frequency (RF) WDM-based passive sensor networks is demonstrated. The proposed solution preserves all the above advantages for sensing interrogation. By using WDM devices with low insertion losses for spectral splitting of a RF modulated broadband light source (BLS) it is possible to enhance the power budget of the network. In Section 2, a brief description of the theoretical background is presented. To test the concept, Section 3 analyzes the performance of an optical intensity sensor system compatible with a CWDM network as well as the self-reference property, thus emulating unexpected losses by means of a variable optical attenuator (VOA). Relative errors of the measurements shown in this section are given. Moreover, a system analysis in terms of optical power budget is studied thus establishing the limits for remote sensor interrogation. Both low-cost Analog-to-Digital converter (ADC) at the reception stage and virtual instrumentation techniques supported on a LabVIEW^®^ platform for developing two virtual delay lines and for controlling the sensor operation are used. Finally, the main conclusions of this work are reported in Section 4.

## 2. Theoretical Background

**Figure 1.**
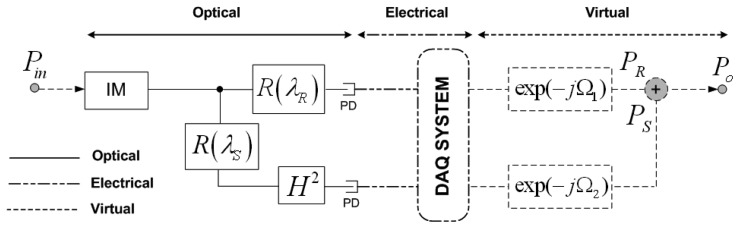
Filter model of the proposed topology for a generic remote sensing point with two virtual delay lines, after acquisition, at the reception stage. IM: Intensity Modulator, PD: Photodetector.

The digital filter schematic for a single fiber-optic sensor topology is illustrated in Figure 1, being *H* the sensor loss modulation. At the reception stage, after signal acquisition, virtual phase-shifts *Ω*_1_ at the reference channel and *Ω*_2_ at the sensing channel are applied, respectively, to the RF modulating signal providing a flexible and easy-reconfigurable operation point of the remote sensor. These delay line filters can be deployed in the optical, electrical or virtual domain but with a coefficient *β* which depends on the optical sensor loss modulation, *H*, in the sensing point. In Figure 1 this sensor loss modulation appears squared due to the reflective operation of the sensing structure, as the light crosses twice the sensor thus providing a sensing system with a doubled sensitivity. For a deeper comprehension, the works reported in References [13,14] are recommended.

Attending to the digital filter model of the sensor topology, the normalized system output, *i.e.*, the transfer function of the system *H_0_* = *P_0_*/*P_in_*, can be directly identified with a digital Finite Impulse Response (FIR) filter in the Z-Transform domain, as shown in Equation (1):

(1) $$H_{0}=\frac{P_{0}}{P_{in}}=\alpha \;\cdot \;(1+\beta \;\cdot \;z^{-1})$$

where *z*=*e*^−j(*Ω*_2_−*Ω*_1_)^, being *α* and *β* defined as follows:

(2) $$\alpha =m_{R}\cdot R\;(\lambda_{R})\cdot d_{R}\cdot e^{-j\cdot \Omega_{1}}$$

(3) $$\beta =\frac{m_{s}\;\cdot \;R\;(\lambda_{s})\;\cdot \;d_{s}}{m_{R}\;\cdot \;R\;(\lambda_{R})\;\cdot \;d_{R}}H^{2}$$

and where *m_R_*, *R*(*λ_R_*)and *d_R_* are the RF modulation index, the reflectivity of the FBG and the photodetector response at the reference wavelength *λ_R_* respectively, and *m_s_*, *R*(*λ_s_*) and *d_s_* are those parameters but for the sensing wavelength *λ_s_*.

Two measurement parameters can then be defined at the remote sensing point. On one hand, the parameter *R* which is given by the ratio between the voltage values received for different delay configurations. And, on the other hand, the output phase *Φ* of the acquired electrical signal, also dependant on the delays configured at the reception stage. Those parameters are given by Equations (4) and (5), respectively:

(4) $$R=\frac{V_{o}(f,\Omega_{2}){|}_{\Omega_{1}=0}}{V_{o}{\;\;(f,\Omega_{1})}_{\Omega_{2}=0}}=\frac{{\left[1+\;(\frac{2\beta }{1+\beta^{2}})\cos\Omega_{2}\right]}^{1/2}}{{\left[1+\;(\frac{2\beta }{1+\beta^{2}})\cos\Omega_{1}\right]}^{1/2}}$$

(5) $$\phi =\arctan\left[\frac{-(\sin\Omega_{1}+\beta \sin\Omega_{2})}{\left(\cos\Omega_{1}+\beta \cos\Omega_{2}\right)}\right]$$

For a fixed value of both the modulation frequency and the delays selection, both measurement parameters of the generic remote sensing point depend only on *β* which is insensitive to external power fluctuations in the optical link. Moreover, both self-referencing parameters can be determined for any pair of values of angular frequencies, *i.e.*, delays, (*Ω*_1_, *Ω*_2_).

## 3. Experimental Setup and Results

**Figure 2.**
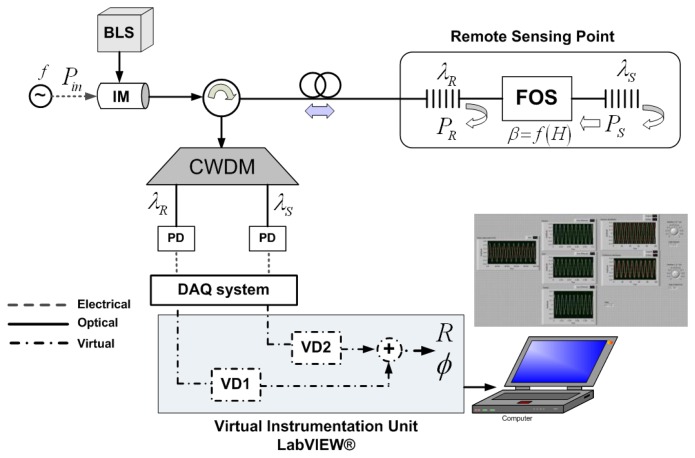
Experimental setup. Inset: user graphical interface for *Φ* parameter. BLS: Broadband Light Source, IM: Intensity Modulator, PD: Photodetector.

The experimental setup is shown in Figure 2, where the topology is performed partially in the optical domain and partially in the digital electronics and virtual domains.

**Figure 3.**
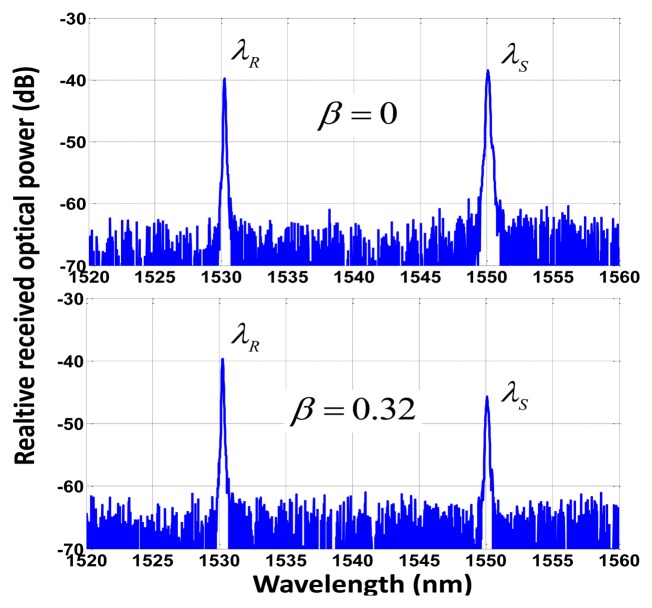
Optical spectrum of both the reference (*λ_R_*) and sensing (*λ_s_*) channels before being sliced through the CWDM demultiplexer device, at different *β* values.

A broadband light source (BLS) modulated at a single frequency *f* = 100 Hz by means of an acousto-optic modulator (IM) is employed to launch optical power into the configuration through the broadband circulator. A 1 km-long SMF feeder fiber connecting the header with the remote sensing point was used for the experiment. A pair of low-cost FBGs is used at the remote sensing point, being placed before and after the FOS, thus obtaining a reference channel and sensing channel, respectively. The latter contains the power modulation induced in the FOS by the measurand. Their central wavelengths are *λ_R_* 1,530.2 *nm* and *λ_s_* 1,550.1 *nm* compatible with standard ITU G.694.2 for CWDM networks, see Figure 3. The optical signal is demultiplexed by a CWDM device and delivered to two different switchable gain InGaAs photodetectors. The Data Acquisition (DAQ) board includes an analog/digital converter (ADC) and performs the signal aggregation while delay line functionality and signal processing are achieved with virtual instrumentation techniques. All these elements are located at the reception stage. DAQ card performs a 14-bit Analog-to-Digital conversion for each analog input and a 48 kS/s of maximum aggregate sampling rate. Finally a PC with LabVIEW^®^ software is used to control the system. The LabVIEW^®^ control panel and user graphical interface for the output phase parameter *Φ* after signal acquisition from the DAQ board can be seen in Figure 2-inset.

**Figure 4.**
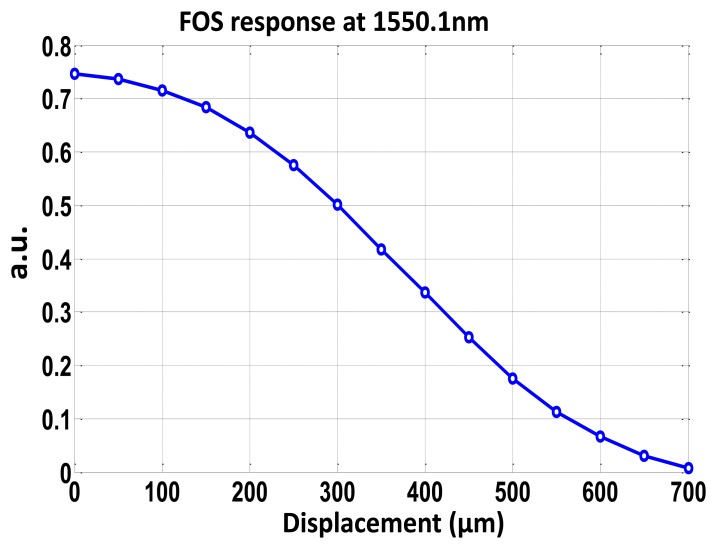
Calibration curve of the sensor loss modulation *H* for the taper-based displacement sensor.

In the reception stage, each signal is compound by 240 samples as a compromise between the performance/limitations of the DAQ board. The displacement sensing system resolution is found to be 14 μm when measuring the output phase, and 2.1 μm (*i.e.*, amplitude resolution of 0.042 dB) when considering the parameter *R*. As shown in Figure 4, a 700 μm input full range (*i.e.*, Full Scale—F.S.) is considered. A better sensor resolution of 0.025 dB but in the same order of magnitude was obtained in the work reported in [16], where erbium doped fiber (EDF) amplification was used to enhance a frequency modulated continuous wave (FMCW) technique for referencing optical intensity sensors. Nevertheless, since the number of samples and the total sampling time provided by the DAQ board is the same, for a lower modulation frequency a better measurand resolution is expected. And if a greater resolution is required in our passive remote sensing topology, a DAQ system with a higher sampling rate should be used, assuming that there is no limitation in terms of optical power budget in our proposed topology. The 0.3% F.S. measurand resolution obtained is better than the values reported in [15], which were around 1.3% F.S., even though lower frequencies are used, of tenths of Hz. Resolution improvement is mainly due to the reflective operation and better *R* parameter performance of our sensing structure.

To test the concept, a SMF-based taper operating as a micro-displacement sensor is placed between each FBG with sensor loss modulation *H*. The taper was obtained by elongation of singlemode fiber during the arc discharge provided by a splicing machine in a semiautomatic fabrication process. Waist cross-section and waist length were 78 μm and 15 mm, respectively. Nevertheless better sensor sensitivities have been demonstrated when decreasing the waist diameter [17], even in combination with FBGs for strain measurements [18]. Once the taper is fixed onto a micro-positioning stage system, the optical loss transmission coefficient of the taper is sensitive to the displacement between the two fiber ends. Figure 4 shows the calibration curve of the sensor loss modulation *H versus* displacement [19].

**Figure 5.**
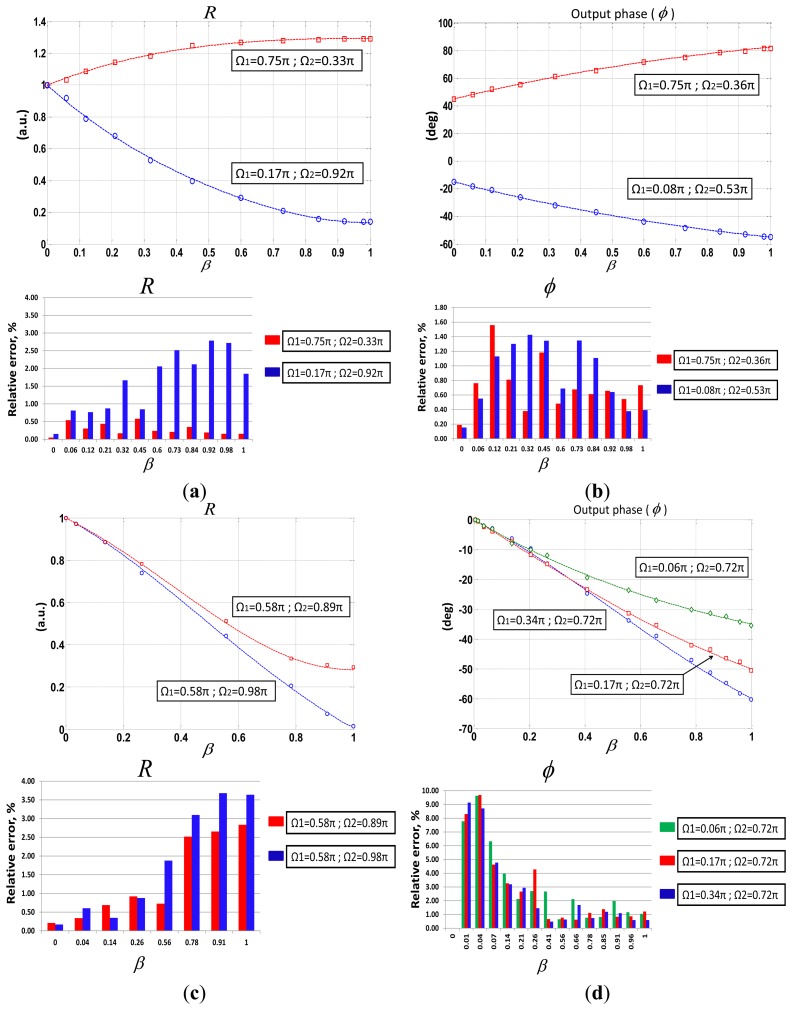
Experimental results and theoretical curves for both self-referencing parameters at different virtual phase-shifts, (**a**) and (**c**) *R* parameter; (**b**) and (**d**) Output phase *Φ*. Theoretical curves are drawn in solid lines. Figures inset: relative errors (in %) of the measurements taken.

Different calibration curves *versus* optical loss modulation of the sensor, *β*, were obtained, for different phase-shifting values. Results showed good agreement between theory and measurements, as can be seen in set of figures comprising Figure 5. Five measurements per each value of *β* were taken for the different phase-shifts selected thus obtaining the mean of the self-referencing parameters. In all cases, relative errors around 1%–3% were obtained. This fact assures good performance to guarantee the sensor interrogation and the self-referencing property. However, it can be seen that when operating at a phase-shift condition nearly *Ω_i_*= ± *π*, *R* measurement stability decreases but not beyond 4%. A similar performance is achieved by using *Φ* parameter with the exception on those cases in which *β*≈0. Nevertheless, phase-shifting configurations could be chosen in order to improve any system feature depending on specific requirements. For instance, the blue curve of Figure 5(d) shows a linear regression coefficient of *r* = 0.9994 quite close to the unit with regards to *R* parameter performance. Phase-shifts requirements for the latter were *Ω*_1_=0.58 *π, Ω*_2_=0.98 *π*, going beyond previous works [19] in which phase-shifts around π radians caused a malfunction in the automation system.

**Figure 6.**
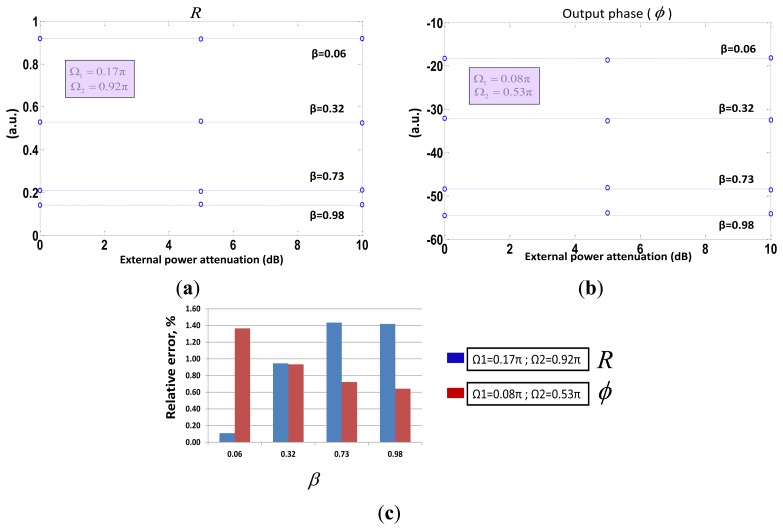
Self-reference test of both measurement parameters *versus* induced power losses (up to 10 dB) for different values of *β*. Figure Inset: relative error (%) of the measurements.

In addition, the self-reference property of both measurement parameters was tested with regards to power fluctuations along the optical system. A singlemode variable optical attenuator (VOA) was located after the broadband circulator thus emulating unexpected power losses, up to 10 dB, in the fiber lead from the optical source to the remote sensing point. In Figure 6 no correlation between the measurements of both self-referencing parameters and the induced power attenuation is shown, as expected.

### 3.1. System Analysis

Considering the measurement results, in the following the optical power budget of the proposed reflective topology is analyzed. Table 1 shows the optical power analysis of the WDM-based remote sensing topology.

The power budget of the system can be calculated using Equation (6):

(6) $$P_{in}\;(\text{dBm})=Att_{\text{AOM}}\;(dB)+Att_{\text{cir}}\;(dB)+\alpha \;(dB/km)\;\cdot \;L(km)+R_{\text{FBG}-\text{ref}}+Att_{\text{FOS}}+R_{\text{FBG}-\text{sens}}+\alpha (dB/km)\;\cdot \;L(km)+Att_{\text{CWDM}}\;(dB)+Att_{\text{con}}\;(dB)+P_{\text{out}}\;(\text{dBm})$$

where *p_in_*(*dbm*) is the optical power launched into the system, *L*(*km*) is the total length of the fiber lead connecting the remote sensing point, and *p_out_*(*dbm*)is the photodiode (PD) sensitivity. The latter is directly related to the PD noise-equivalent power (NEP) figure of merit and the value provided in Table 1 refers to the worst case, in terms of both NEP and bandwidth.

Computing Equation (6) at both reference and sensing channels, and assuming an optical attenuation in singlemode fiber of 0.2 dB/km, *α*(*dbm*/*km*), in the C-band as well as seven adapters within the link, a maximum length of 16 km could be obtained. Worst case was obtained when evaluating the sensing channel (λ = 1,550 nm). It is worth noticing that with 99% reflectance FBG at 1,550 nm, the reach could be extended up to 23 km.

## 4. Conclusions

In this work, a single radio-frequency self-referenced CWDM intensity-based fiber-optic sensor using virtual delay lines at the reception stage is presented. The sensor topology operates in reflective configuration and takes advantage of the use of FBGs and CWDM devices. It also allows a high scalability and an enhancement of the power budget as CWDM devices with low insertion losses are used for spectral splitting. With the proposed topology, and considering the available optical power provided by the optical source, a remote sensing distance of 16 km could be obtained. Improving the reflectivity of the FBG at the sensing channel a maximum reach of 23 km is estimated. The proposed virtual configuration avoids the deployment of physical delay lines, either in the optical and electrical domains, thus allowing an even more compact, cost-effective and easy-reconfigurable solution, through two delays, for sensor operation while keeping all the advantages of optical sensing. In addition, there is no need for a lock-in amplifier at the reception stage. Assuming no optical power budget limitation, system resolution and number of channels to be multiplexed are limited by DAQ board sampling rate and its ADC resolution, but easily improved. A micro-displacement sensor is used and two different self-referencing parameters have been tested. This solution can lead either to a higher automation in the interrogation of future WDM-based remote sensor networks or to a lower cost. It can also improve scalability and automation in monitoring system [20] of fault location or drop fibers optical power losses in WDM passive optical network (PON) architectures.

## Figures and Tables

**Table 1.** Optical power budget analysis of the proposed CWDM-based remote sensing scheme.

	BLS output power, *P_in_*	PD sensitivity (dBm)	**Device, Insertion Loss**					

			IM, *Att_AOM_* (dB)	Circulator, *Att_cir_* (dB)	CWDM demux, *Att_CWDM_* (dB)	Adapters *, *Att_con_* (dB)	FBG (reflectivity), *R_FBG_*_−_*_i_* (dB)	FOS^1^, *Att_FOS_*(dB)
1,530 nm (ref)	−15 dBm	−60.5	10	0.6	1.8	0.5	3.4 (46%)	17
1,550 nm (sens)	−17.5 dBm						3 (50%)	

* losses per connector;

1 measured at maximum input Full-Scale (F.S), worst case (β = 1).

## References

[1] Montalvo J., Frazao O., Santos J.L., Vazquez C., Baptista J.M. (2009). Radio-frequency self-referencing technique with enhanced sensitivity for coarse WDM fiber optic intensity sensors. J. Lightwave Technol..

[2] Leandro D., Ullán A., Loayssa A., López-Higuera J.M., López-Amo M. (2011). Remote (155 km) fiber bragg grating interrogation technique combining Raman, Brillouin, and erbium gain in a fiber laser. IEEE Photon. Tech. Lett..

[3] Saitoh T., Nakamura K., Takahashi Y., Iida H., Iki Y., Miyagi K. (2007). Ultra-long-distance fiber bragg grating sensor system. IEEE Photon. Tech. Lett..

[4] Murtaza G., Senior J.M. (1994). Methods for providing stable optical signals in dual wavelength referenced LED based sensors. IEEE Photon. Tech. Lett..

[5] Wang A., Xiao H., Wang J., Wang Z., Zhao W., May R.G. (2001). Self-calibrated interferometric intensity-based optical fiber sensors. J. Lightwave Technol..

[6] Baptista J.M., Abad S., Rego G.M., Ferreira L.A., Araújo F.M., Santos J.L., Lage A.S. (2004). Wavelength multiplexing of frequency-based self-referenced fiber optic intensity sensors. Opt. Eng..

[7] Baptista J.M., Santos J.L., Lage A.S. (2000). Self-referenced fibre optic intensity sensor based on a multiple beam Sagnac topology. Opt. Commun..

[8] Dong X., Tam H.Y., Shum P. (2007). Temperature-insensitive strain sensor with polarization maintaining photonic crystal fiber based Sagnac interferometer. Appl. Phys. Lett..

[9] Vázquez C., Montalvo J., Montero D.S., Pena J.M.S. (2006). Self-referencing fiber-optic intensity sensors using ring resonators and fiber bragg gratings. IEEE Photon. Tech. Lett..

[10] Spillman W.B., Lord J.R. (1987). Self-referencing multiplexing technique for fiber-optic intensity sensors. J. Lightwave Technol..

[11] Caucheteur C., Mussot A., Bette S., Kudlinski A., Douay M., Louvergneaux E., Mégret P., Taki M., González-Herráez M. (2010). All-fiber tunable optical delay line. Opt. Express.

[12] Abad S., López-Amo M., Araújo F.M., Ferreira L.A., Santos J.L. (2002). Fiber Bragg grating-based self-referencing technique for wavelength-multiplexed intensity sensors. Opt. Lett..

[13] Montalvo J., Araujo F.M., Ferreira L.A., Vazquez C., Baptista J.M. (2008). Electrical FIR filter with optical coefficients for self-referencing WDM intensity sensors. IEEE Photon. Tech. Lett..

[14] Montero D.S., Vázquez C., Baptista J.M., Santos J.L., Montalvo J. (2010). Coarse WDM networking of self-referenced fiber-optic intensity sensors with reconfigurable characteristics. Opt. Express.

[15] Fernandes A.J.G., Jesus C., Jorge P.A.S., Baptista J.M. (2011). Fiber optic intensity sensor referenced with a virtual delay line. Opt. Commun..

[16] Pérez-Herrera R.A.F., Frazao O., Santos J.L., Araújo F.M., Ferreira L.A., Baptista J.M., López-Amo M. (2009). Frequency modulated continuous wave system for optical fiber intensity sensors with optical amplification. IEEE Sens. J..

[17] Arregui F.J., Matías I.R., López-Amo M. (2000). Optical fiber strain gauge based on a tapered single-mode fiber. Sens. Actuators A Phys..

[18] Frazão O., Silva S.O., Guerreiro A., Santos J.L., Ferreira L.A., Araújo F.M. (2007). Strain sensitivity control of fiber Bragg grating structures with fused tapers. Appl. Opt..

[19] Montero D.S., Vázquez C. (2012). Interrogation of remote intensity-based fiber-optic sensors deploying delay lines in the virtual domain. Proc. SPIE.

[20] Montalvo J., Montero D.S., Vázquez C., Baptista J.M., Santos J.L. (2010). Radio-frequency self-referencing system for monitoring drop fibres in wdm passive optical networks. IET Optoelectron..

